# The Italian Unitary Society of Colon-proctology (SIUCP: Società Italiana Unitaria di Colonproctologia) guidelines for the management of anal fissure

**DOI:** 10.1186/s12893-023-02223-z

**Published:** 2023-10-13

**Authors:** Antonio Brillantino, Adolfo Renzi, Pasquale Talento, Francesca Iacobellis, Luigi Brusciano, Luigi Monaco, Domenico Izzo, Alfredo Giordano, Michele Pinto, Corrado Fantini, Marcello Gasparrini, Michele Schiano Di Visconte, Francesca Milazzo, Giovanni Ferreri, Andrea Braini, Umberto Cocozza, Massimo Pezzatini, Valeria Gianfreda, Alberto Di Leo, Vincenzo Landolfi, Umberto Favetta, Sergio Agradi, Giovanni Marino, Massimilano Varriale, Massimo Mongardini, Claudio Eduardo Fernando Antonio Pagano, Riccardo Brachet Contul, Nando Gallese, Giampiero Ucchino, Michele D’Ambra, Roberto Rizzato, Giacomo Sarzo, Bruno Masci, Francesca Da Pozzo, Simona Ascanelli, Fabrizio Foroni, Alessio Palumbo, Patrizia Liguori, Angela Pezzolla, Luigi Marano, Antonio Capomagi, Eugenio Cudazzo, Francesca Babic, Carmelo Geremia, Alessandro Bussotti, Mario Cicconi, Antonia Di Sarno, Federico Maria Mongardini, Antonio Brescia, Leonardo Lenisa, Massimiliano Mistrangelo, Maria Laura Sandoval Sotelo, Luciano Vicenzo, Antonio Longo, Ludovico Docimo

**Affiliations:** 1 Deparment of Surgery, “A. Cardarelli” Hospital, Via A. Cardarelli 9, Naples, 80131 Italy; 2 “Buonconsiglio-Fatebenefratelli” Hospital, Naples, Italy; 3 Department of Surgery, Pelvic Floor Center, AUSL-IRCCS Reggio Emilia, Reggio Emilia, Italy; 4 Department of General and Emergency Radiology, “A. Cardarelli” Hospital, Naples, Italy; 5 Department of Advanced Medical and Surgical Sciences, University of Campania “L. Vanvitelli”, Naples, Italy; 6 “Pineta Grande” Hospital, “Villa Esther” Clinic, Avellino, Italy; 7 Department of General and Emergency Surgery, AORN dei Colli Monaldi-Cotugno-CTO, CTO Hospital, Naples, Italy; 8 Department of General and Emergency Surgery, University of Salerno, Hospital of Mercato San Severino, Salerno, Italy; 9 Humanitas Castelli, Bergamo, Italy; 10 Department of Surgery, “Dei Pellegrini” Hospital, ASL Napoli 1, Naples, Italy; 11 Oncologic Colorectal Unit, “Sant’Andrea” University Hospital, Rome, Italy; 12 Department of General Surgery, Colorectal and Pelvic Floor Diseases Center, “Santa Maria Dei Battuti” Hospital, Conegliano, TV Italy; 13 Department of General Surgery, Azienda Sanitaria Friuli Occidentale (ASFO), Pordenone, Italy; 14 Department of General Surgery, “S. Maria Degli Angeli” Hospital, Putignano (Bari), Italy; 15 “Dei Castelli” Hospital, ASL Roma 6, Rome, Italy; 16 Unit of Colonproctologic and Pelvic Surgery, “M.G. Vannini” Hospital, Rome, Italy; 17 Department of General and Minivasive Surgery, “San Camillo” Hospital, Trento, Italy; 18 Department of General and Specalist Surgery, AORN “S.G. Moscati”, Avellino, Italy; 19 Unit of Proctology and Pelvic Surgery, “Città di Pavia” Clinic, Pavia, Italy; 20 Humanitas Gavazzeni/Castelli Bergamo, Bergamo, Italy; 21 Department of General Surgery, “Santa Marta e Santa Venera” Hospital of Acireale, Catania, Italy; 22 Department of General and Emergency Surgery, “Sandro Pertini” Hospital, Asl Roma 2, Rome, Italy; 23 “La Sapienza” University of Rome, “Umberto1” Polyclinic, Rome, Italy; 24 General Ad Urgent Surgery, “U. Parini” Regional Hospital, Aosta Valley, Italy; 25 Unit of Proctologic Surgery, “Sant’Antonio” Clinic, Cagliari, Italy; 26 Unit of General Surgery, “Maggiore” Hospital, Bologna, Italy; 27 Department of General and Oncologic-Minivasive Surgery, “Federico II” University, Naples, Italy; 28 Department of General Surgery, Hospital of Conegliano AULSS 2, Marca Trevigiana, Treviso, Italy; 29 Department of General Surgery, University of Padova, “Sant’Antonio” Hospital, Padova, Italy; 30 “San Carlo di Nancy” Hospital, Rome, Italy; 31 Department of Surgery, “Santa Maria dei battuti” Hospital, San Vito al Tagliamento, Pordenone, Italy; 32 Department of Surgery, University Hospital of Ferrara, Ferrara, Italy; 33 Department of Surgery, “Mater Dei” Hospital, Bari, Italy; 34 Department of Surgery, University Aldo Moro, Bari, Italy; 35 Academy of Applied Medical and Social Sciences - AMiSNS: Akademia Medycznych i Spolecznych Nauk Stosowanych, Elbląg, Poland; 36 “Montanari” Hospital, Morciano di Romagna, Rimini, Italy; 37 Department of Surgery, Hospital of Cattinara, ASUGI Trieste, Trieste, Italy; 38 “San Carlo Di Nancy” Hospital, Rome, Italy; 39 Department of General Surgery, “Sant’Omero-Val Vibrata” Hospital, Teramo, Italy; 40 Department of Oncologic Colorectal Surgery, University Hospital S. Andrea, “La Sapienza” University, Rome, Italy; 41 Department of Surgery, Humanitas San Pio X, Surgery Unit, Pelvic Floor Centre, Milano, Italy; 42 Department of Surgery, “Le Molinette” Hospital, Torino, Italy; 43 “Madonna Della Fiducia” Clinic, Rome, Italy

**Keywords:** Anal fissure, Anal spasm, Endoanal ultrasound, Anal manometry, Anal dilatation, Sphincterotomy, Fissurectomy, SIUCP

## Abstract

**Introduction:**

The aim of these evidence-based guidelines is to present a consensus position from members of the Italian Unitary Society of Colon-Proctology (SIUCP: Società Italiana Unitaria di Colon-Proctologia) on the diagnosis and management of anal fissure, with the purpose to guide every physician in the choice of the best treatment option, according with the available literature.

**Methods:**

A panel of experts was designed and charged by the Board of the SIUCP to develop key-questions on the main topics covering the management of anal fissure and to performe an accurate search on each topic in different databanks, in order to provide evidence-based answers to the questions and to summarize them in statements. All the clinical questions were discussed by the expert panel in different rounds through the Delphi approach and, for each statement, a consensus among the experts was reached. The questions were created according to the PICO criteria, and the statements developed adopting the GRADE methodology.

**Conclusions:**

In patients with acute anal fissure the medical therapy with dietary and behavioral norms is indicated. In the chronic phase of disease, the conservative treatment with topical 0.3% nifedipine plus 1.5% lidocaine or nitrates may represent the first-line therapy, eventually associated with ointments with film-forming, anti-inflammatory and healing properties such as Propionibacterium extract gel. In case of first-line treatment failure, the surgical strategy (internal sphincterotomy or fissurectomy with flap), may be guided by the clinical findings, eventually supported by endoanal ultrasound and anal manometry.

## Preliminary statement

The Italian Unitary Society of Colon-Proctology (SIUCP: Società Italiana Unitaria di Colon-Proctologia) was founded with the aim of implementing the quality of patients care through the employment of new technologies and the support of scientific research. The designed Committee for developing the SIUCP guidelines is composed of society members who showed particular expertise in the colon-proctologic diseases and stood out in the related scientific research.

These guidelines were formulated to provide detailed informations for all health-care workers and patients about the main topics concerning the diagnosis and treatment of anal fissure, and, consequently, to guide the physicians in the choice of the best treatment option, according with the available literature.

However, these guidelines are not impositive of specific treatments, nor inclusive of all the adequate diagnostic and therapeutic options and, therefore, do not exclude that the same results can be obtained with other not mentioned methods of care.

In the clinical practice, the final decision of adopting a specific diagnostic or therapeutic choice, should be made by physician, according with each patients individual characteristics.

Therefore, every physician may deviate from these guidelines whenever it is deemed appropriate, in relation to the specific clinical case, the circumstances presented by the single patient and the available resources.

## Background

Anal fissure is a common proctologic disease, accounting for up to 10% of anorectal complaints in specialty clinics [1]. It represents an oval or tear-shaped ulceration of anal canal extending from the dentate line to the anal verge [2–8]. In up to about 80–90% of cases it occurs solitary in the posterior midline and more rarely in the anterior one. The anterior fissures are relatively more frequent in women and are common in postpartum [4].

Fissures occurring away from midline and multiple fissures are considered atypical and tend to be associated with other diseases including human immunodeficiency virus (HIV) infection, syphilis, tuberculosis, trauma and ano-receptive practices, psoriasis, Crohn’s disease and malignancy [2, 3].

Anal fissures are commonly divided, temporally and morphologically, in acute and chronic. Acute anal fissures have been present for less than 6 weeks and appear as a superficial and longitudinal tears with demarcated edges. Chronic anal fissures have been present for more than 6 weeks and show one or more signs of chronicity including an external sentinel skin tag at the external apex, a hypertrophied anal papilla at the internal apex, raised or heaped-up edges and visibility of the concentrically oriented white fibers of the internal sphincter muscle at the base of the fissure [2–7].

The etiopathogenesis of the anal fissure is still unclear and probably recognizes a multifactorial origin. The fissure typically occurs after a trauma of the anoderm caused by passage of hard stool or by irritation of diarrhea. The higher prevalence of the posterior location could be explained by the greater mechanical stress caused by the anorectal angle posteriorly. According with studies showing an increased anal tone and a reduction in posterior anoderma vascular blood flow in patients with anal fissures [9, 10], it has been speculated that the increased sphincter tone caused by the intense anal pain typical of fissuring, predisposes the mucosa to ischemia and impedes the healing of the fissure, generating a vicious cycle [4].

The typical clinical presentation includes a severe painful symptomatology occurring during defecation and persisting for hours afterwards, eventually associated with slight bleeding. Occasionally, especially in women, the main reported complaint is bleeding, rather than pain. An asymptomatic fissure should raise the suspicion of Crohn’s disease [4].

Diagnosis of anal fissure can be strongly suggested by patient history and can be confirmed, in the majority of cases, by direct visualization of fissure through divarication of buttocks and during straining with the patient in the left lateral or prone position [2, 3]. Differential diagnosis includes abscess, external hemorrhoid thrombosis, anal cancer, pruritus ani and a variety of anogenital infections. If fissure is not readily apparent, it can be highly suspected, at digital anorectal examination, in presence of typical findings including anal sphincter hypertonia, tenderness in the posterior midline, external “sentinel” skin tag and internal hypertrophied anal papilla. In this setting, anoscopy is not advisable because may cause significant pain and does not allow an adequate anoderma visualization. In case of suspected diagnosis, generally, empiric treatment and subsequent re-examination is appropriate [1]. Instead, in case of doubtful diagnosis, suspected abscess or thrombosed hemorrhoids, multiple anal fissures or painless anal fissure unresponsive to medical therapy, examination under anesthesia with eventual biopsy and cultures is advocated [11].

## Methods

A panel of experts was designed and charged by the Board of the Italian Unitary Society of Colonproctology (SIUCP: Società Italiana Unitaria di Colonproctologia) to develop key-questions on the main topics covering the diagnosis and treatment of anal fissure. Then, leading specialists in this field, guided by a central coordinator, performed an accurate search on each topic in different databanks (MEDLINE, SCOPUS, EMBASE) in order to provide evidence-based answers to the questions and to summarize them in statements. The search strategy was based on the following key-words combinations: “anal fissure and diagnosis”, “anal fissure and imaging”, “anal fissure and ultrasound”, “anal fissure and sepsis”, “anal fissure and abscess”, “anal fissure and fistula”, “anal fissure and manometry”, “anal fissure and treatment”, “anal fissure and therapy”, “anal fissure and fiber”, “anal fissure and nifedipine”, “anal fissure and nytroglicerine”, “anal fissure and glycerin trinitrate”, “anal fissure and diltiazem”, “anal fissure and metronidazole”, “anal fissure and botulinum”, “anal fissure and dilatation”, “anal fissure and sphincterotomy”, “anal fissure and fissurectomy”, “anal fissure and flap”, “anal fissure and tibial nerve stimulation”. Basing on this search, 677 papers were screened for inclusion and, of these, 293 were excluded, being represented by cases series, case reports, letters to the Editor, proceedings, studies without abstracts or addressing incorrect topic. Of 384 full-text articles assessed for eligibility, 249 were excluded because higher-level evidence studies were available. Consequently, 135 studies were analyzed to provide evidence-based answers to each key-question. Complexively, the references of this manuscript were represented by 140 entries including 135 studies arising from the mentioned research and 5 additional records comprehending articles and book chapters addressing the epidemiology, pathogenesis and clinical presentation of anal fissure.

All the clinical questions were discussed by the expert panel in different rounds through the Delphi approach [12] and, for each statement, a consensus among the experts was reached. The central coordinator assembled the different answers derived from each round and, with the cooperation of the expert panel, prepared the definitive guidelines, resulting in the present manuscript. All the experts contributed to the development of current guidelines and the manuscript was reviewed and approved by all the authors. The questions were created according to the PICO (Patients, Intervention, Comparison, Outcome) criteria, and the statements developed adopting the GRADE (Grading of Recommendations, Assessment, Development, and Evaluations) methodology [13–15] (Table 1). In case of relevant topics with undetectable quality of evidence due to lack of pertinent studies, the related statements were based on the expert panel opinion.

**Table 1:** Grading of recommendations according to GRADE system

**Grade**	**Description**	**Benefit vs risks**	**Quality of studies**	**Implications**
1A	Strong recommendation, high-quality evidence	Benefits clearly outweigh risks and burdens or vice versa	RCTs without important limitations or overwhelming evidence from observational studies	Strong recommendation; can apply to most patients in most circumstances without reservation
1B	Strong recommendation, moderate-quality evidence	Benefits clearly outweigh risks and burdens or vice versa	RCTs with important limitations or exceptionally strong evidence from observational studies	Strong recommendation; can apply to most patients in most circumstances without reservation
1C	Strong recommendation, low or very low-quality evidence	Benefits clearly outweigh risks and burdens or vice versa	Observational studies or case series	Strong recommendation but may change when higher-quality evidence becomes available
2A	Weak recommendation, high-quality evidence	Benefits closely balanced with risks and burdens	RCTs without important limitations or overwhelming evidence from observational studies	Weak recommendation; best action may differ depending on circumstances or patients’ or societal values
2B	Weak recommendation, moderate-quality evidence	Benefits closely balanced with risks and burdens	RCTs with important limitations or exceptionally strong evidence from observational studies	Weak recommendation; best action may differ depending on circumstances or patients’ or societal values
2C	Weak recommendation, low or very low-quality evidence	Uncertainty in the estimates of benefits, risks, and burdens; benefits, risks, and burdens may be closely balanced	Observational studies or case series	Very weak recommendation; other alternatives may be equally reasonable

## Questions and statements

### In patients with anal fissure, which are the appropriate morphological investigations?

- *According with the available scarce literature, no recommendation can be made concerning the employment of imaging investigations in patients with typical acute anal fissure, especially in presence of anal pain and spasm that make challenging to perform any endoanal examination.*
- *In patients with atypical anal fissures, especially when an associated pathology including inflammatory bowel diseases or colorectal and anal cancer is suspected, imaging investigations such as colonscopy, anoscopy and endoanal ultrasound ma be useful for diagnostic purpose (weak recommendation based on low-quality evidence, 2C).*
- *In patients with chronic anal fissure poor responsive to medical therapy, in order to evaluate the presence of an associated occult anal sepsis, a morphological evaluation with endoanal ultrasound may be considered (weak recommendation based on low-quality evidence, 2C).*
- *In patients with chronic anal fissure and suspected occult local sepsis, if endoanal ultrasound is not available, Magnetic Resonance Imaging may be considered as alternative diagnostic tool (experts opinion)*

Commonly, in patients with typical acute anal fissure, imaging investigations are not necessary nor advisable and usually not feasible in the setting of severe anal pain associated with internal sphincter hypertonia. In case of atypical anal fissure, uncertain diagnosis and/or futures suggestive of secondary anal fissure and/or significant bleeding in patients over 50 years of age or with increased risk of colorectal cancer, imaging investigations including endoscopy and endoanal ultrasound may be required, depending on the suspected underlying disease [16]. Particularly, if an occult anal sepsis is suspected in patients with chronic anal fissure poor responsive to medical therapy, an endoanal ultrasound examination may be considered, since the existing scarce literature suggests in these cases an associated local sepsis in a percentage variable between 5 and 65%, depending on whether the sepsis has been identified during surgery or by preoperative imaging evaluation.

As early as 1948, Whitney reported that some of his patients with “cryptitis” had an associated anal fissure [17]. Subsequently, Parks in the 1973 described a series of 33 patients with intersphincteric abscess, recognizing an associated anal fissure in eight (24.2%) of them [18]. Afterwards, Gupta et al. reported, among 532 patients treated for chronic anal fissures, as intra-operative findings, 88 (16.5%) cases of associated suppurative pathologies including an abscess in 42%, a fistula in 39% and a seroma in 19% of them [19]. More recently, Naldini et al., in a prospective series of 172 patients with chronic anal fissure evaluated by preoperative endoanal ultrasound, described an associated abscess in 117 (65%) cases, with expression of 91 (52.9%) intersphincteric and 21 (12.2%) low transphincteric fistulas [20]. According with these results, the authors speculated that chronic fissures may persist because of hiding sepsis in the anal canal and that anal fissure chronicity might be the clinical and pathological expression of a coexisting intersphincteric or low transphincteric fistula, as showed by endoanal ultrasound. However, in the same study, these impressive endosonographic findings were not compared with the intraoperative ones, therefore questioning the accuracy of the results of preoperative endoanal ultrasound evaluation. Finally, in a recent large series of 988 patients undergone surgical treatment for chronic anal fissure, an associated local sepsis was intraoperatively found in 55 (5.5%) cases, including 23 (42%) abscesses and 32 (58%) fistulas, of whom, 17 were inter-sphincteric and 15 low trans-sphincteric [21].

Overall, the available literature suggests that endoanal ultrasound may detect, in a considerable percentage of patients with chronic anal fissure, the presence of an associated occult sepsis. However, the prevalence of associated sepsis in patients with anal fissure such as the actual impact of endoanal ultrasound on the management of anal fissure are still unclear.

### In patients with anal fissure, which are the appropriate functional investigations?

- *According with the available literature, no recommendation can be made regarding the use of functional investigations such as ano-rectal manometry in patients with acute anal fissure. Commonly, anorectal physiology testings are not routinely performed at this juncture and the initial evaluation of sphincter hypertonia in patients with anal fissure could be based on clinical examination (experts opinion).*
- *In patients with chronic anal fissure poor responsive to medical therapy, in order to accurately select the patients without internal sphincter hypertonia, an ano-rectal manometric evaluation may be considered (weak recommendation based on low-quality evidence, 2C).*
- *Although anal manometry could detect the anal tone more accurately than digital rectal examination, this functional investigation is not always possible in patients with hyperalgesic chronic fissure. In case of not feasibility or availability of manometry, the evaluation of anal tone with digital examination may be considered sufficient (experts opinion).*

The existence, in the majority of patients with anal fissure, of a raised resting pressure profile of anal canal as result of an internal sphincter hypertonia, has been confirmed by many authors and included in the pathogenesis of disease [22–30]. Nevertheless, a subgroup of patients, especially those with anterior and lateral fissures, may show normal pressures of the anal canal, probably reflecting a different etiopathogenesis of the fissure [22, 31–33]. In these subjects, the therapeutic surgical strategy, after failure of medical treatment, is a challenge, because internal sphincterotomy and anal dilatation may induce anal hypotonia and a potential increased risk of postoperative incontinence [34]. Consequently, the question arises on the opportunity to perform a preoperative ano-rectal manometry in patients with chronic anal fissure poor responsive to medical therapy, in order to accurately select the subjects eligible for surgical procedures interfering with anal sphincter system integrity.

Jones et al. prospectively investigated the ability of surgeons to clinically detect the anal tone in 40 consecutive patients with chronic anal fissure, comparing the results of ano-rectal manometry with digital rectal examination undertaken by a surgeon blinded to the manometric findings [35]. As result, clinical assessment of anal tone correctly identified 93% of patients with high manometric maximum resting pressure, yet detected only 16% of those with normal or low pressures, with a positive predictive value of clinical assessment of 40 percent and a negative predictive value of 80 percent. The authors concluded that ability of surgeons to identify patients without anal hypertonia is poor and suggested to selectively investigate by manometry those patients who fail medical therapy, before considering internal sphincterotomy [35].

Similarly, a retrospective study conducted on 100 patients with chronic anal fissure of the posterior commissure showed a complete concordance of digital rectal examination with ano-rectal manometry in detecting a very high anal tone (defined at manometry as a mean anal resting pressure > 101 mmHg) and, conversely, a considerable discordance between digital rectal examination and anal manometry in detecting a normal anal tone (defined as mean anal resting between 45 and 85 mmHg) or a mild augmented anal tone (defined as mean anal resting between 86 and 100 mmHg) [36]. According with these results, the authors highlighted the role of anal manometry in identifying patients at high risk of post-operative complications and in planing a saving sphincter procedure [36].

Anyway, Prohm et al., comparing the outcome after internal sphincterotomy between patients with preoperative manometric findings of increased and normal anal resting pressure, found no significative difference in the prevalence of postoperative fecal incontinence between the two groups, even if in patients with normal preoperative resting pressure the prevalence of postoperative incontinence was higher (3.2% vs 0.7%) [37]. In light of these results, the authors, although admitted that manometry could be useful in selecting patients with chronic anal fissure associated with decreased resting pressure, ultimately questioned the impact of preoperative anal manometry on the postoperative outcome of patients undergoing sphincterotomy [37].

Overall, the scarce existing literature suggests that in patients with chronic anal fissure ano-rectal manometry can detect the anal tone more accurately than digital rectal examination, guiding the surgeon in the choice of the more appropriate surgical treatment. However, the impact of preoperative manometry on the outcome of anal fissure surgery is still unclear and object of debate.

### In patients with acute anal fissure what is the treatment of choice?

- *In patients with acute anal fissure, non-operative management should be considered as the first-line treatment whereas surgical treatment may be considered in the chronic phase, in patients unresponsive after at least 6 weeks conservative treatment (strong recommendation based on moderate quality evidences,1B).*
- *Non operative management in patients with acute anal fissure should include warm sitz baths and increased fiber and water dietary intake up to obtain soft stools (strong recommendation based on moderate quality evidences,1B).*
- *In case of persisting hard stools, fiber supplements and bulk forming laxatives may be added to therapy (expert opinion)*
- *In patients with acute anal fissure, the additional therapy with topical application of sphincter muscle relaxers such as calcium channel blockers and, particularly, 0.3% nifedipine plus 1.5% lidocaine may be considered in case of poor patients adherence to dietary and behavioral medical prescriptions (weak recommendation based on low-quality evidence, 2C).*
- *The integration of topical metronidazole in the non operative management of acute anal fissure may be considered (weak recommendation based on low-quality evidence, 2C).*
- *The additional use of the common analgesic drugs, topical anesthetics and ointments with thermogenic and myorelaxant effect in the treatment of acute anal fissure is reasonable in case of inadequate pain control (experts opinion).*
- *In case of hyperalgesic acute anal fissure not responsive to common painkillers and ointments, a surgical approach in emergency setting may be considered (experts opinion)*
- *Self-induced gradual mechanical anal dilatation with dedicated plastic dilators is commonly suggested and prescribed to patients with anal sphincter hypertonia and spasm. However, due to lack of relevant literature, no recommendations concerning this treatment in patients with anal fissure can be made.*

Current knowledge on the treatment of acute anal fissure mainly arises from 2 historical studies of Jensen SL [38, 39]. In the first study, 103 patients with an acute first episode of posterior anal fissure were randomized to receive a 3 week trial of lidocaine ointment (*n* = 33) versus hydrocortisone ointment (*n* = 35) or warm sitz baths combined with an intake of unprocessed bran (*n* = 35). As result, symptomatic relief was the same regardless of the treatment regimen whereas the healing rate was higher in patients treated with warm sitz baths and bran (87%) if compared with hydrocortisone (82.4%) or lidocaine ointment (60%) [38]. The specific role of warm sitz baths was evaluated in a more recent randomized trial comparing patients treated or not with sitz baths for 4 weeks in addition to oral psyllium husk [40]. The study results suggested that treatment with warm sitz baths improved patient satisfaction without determining a significant increase of healing rate and pain relief [40].

In a second double-blind, placebo-controlled trial, Jensen SL evaluated the effect of unprocessed bran in a dose of 5 g three times daily and a dose of 2.5 g three times daily for 1 year on the recurrence rate of anal fissures, showing significantly fewer recurrences in patients receiving bran 5 g (recurrence rate 16%), when compared with patients receiving bran 2.5 g (recurrence rate 60%; P less than 0.01) and with patients receiving placebo (recurrence rate 68%; P less than 0.01) [39].

The anal fissure healing rate after conservative treatment seems to decrease with the increase of symptoms duration as indicated by a prospective study of 60 patients, showing a 100% healing rate in patients with symptoms duration of < 1 month, compared to 33.3% healing rate in patients with symptoms duration of > 6-months [41].

The literature concerning the topical treatment of acute anal fissure is scarce. A randomized controlled trial compared 141 patients treated with topical 0.2% nifedipine gel every 12 h for 3 weeks with 142 patients receiving topical 1% lidocaine plus 1% hydrocortisone acetate gel, showed a significative higher percentage of remission (95% vs 50%: *p* < 0.01) in nifedipine-treated patients [42]. Unfortunately, these data have still not been confirmed in a context of a multicentric study and, additionally, the ointment with exclusive 0.2% nifedipine is currently not commercially available in Italy.

A single-centre non controlled study evaluated the safety and efficacy of topical treatment of acute anal fissure with 0.5% nifedipine t.i.d for 8 weeks, showing a 85.2% success rate. However, during therapy, patients were encouraged to follow a high-fiber diet, therefore raising the question of the diet influence on the success rate [43].

A retrospective study on a pediatric population with acute anal fissure evaluated the safety and efficacy of 4-weeks topical treatment with 0.3% nifedipine plus 1.5% lidocaine ointment, showing a 93.4% success rate without side effects [44].

Three single-centre randomized controlled trials investigated the role of topical metronidazole in the treatment of acute anal fissure and concluded that the adjunct of topical metronidazole to local treatment with diltiazem, glyceryl trinitrate or lidocaine was associated with a significative increase of healing rate, shorter healing time and lower duration and severity of pain [45–47]. However, these studies had some methodological limitations and their results have still not been confirmed by well-done multi-center randomized controlled trials.

No one study specifically addressed the role of the common analgesic drugs, topical anesthetics and ointments with thermogenic and myorelaxant effect as additional treatment in the non operative management of acute anal fissure. However, in the clinical practice, these drugs are widely and effectively used for the treatment of anal pain caused by the fissure. For this reason, despite the lack of evidence, the expert panel considered reasonable the adjunctive therapy with these drugs in case of inadequate pain control. Finally, in clinical practice, self-induced gradual mechanical anal dilatation is commonly suggested and prescribed to patients with anal stenosis, hypertonia and spasm. However, studies evaluating the outcome of this treatment in patients with anal fissure are lacking, making it difficult to make any pertinent recommendation.

### In patients with chronic anal fissure what is the first-line treatment?

- *In patients with chronic anal fissure and typical clinical presentation (intense anal pain associated with suspected anal sphincter hypertonia at physical examination) the first-line treatment may be represented by topical application of calcium channel blockers or nitrates (0.4% glyceryl trinitrate) (strong recommendation based on moderate-quality evidence, 1B).*
- *The topical use of calcium channel blockers is associated with similar effectiveness and fewer side effects, if compared with nitrates. (strong recommendation based on moderate-quality evidence, 1B).*
- *In patients with chronic anal fissure and typical clinical presentation, topical 0.3% nifedipine plus 1.5% lidocaine may be considered as first-line therapy (weak recommendation based on moderate-quality evidence, 2B).*
- *The adjunctive use of topical ointments with healing properties in the treatment of chronic anal fissure may be reasonable in association with topical calcium channel blockers and nitrates in case of anal sphincter hypertonia or as exclusive treatment in case of anal sphincter hypotonia (experts opinion).*
- *Among the topical ointments with film-forming, anti-inflammatory and healing properties, Propionibacterium extract gel (PeG) may be considered (weak recommendation based on moderate-quality evidence, 2B).*

According with a Cochrane meta-analysis of 18 randomized trials [48], with a multi-center double-blind placebo-controlled trial of 200 patients with chronic anal fissure [49] and with a systematic review [50], the treatment with topical nitroglycerine is associated with an healing rate of about 50%. Moreover, the treatment is limited by occurring of headache in at least 30% of patients, leading to cessation of therapy in up to 20% of them [50–52].

In a 2013 systematic review of 7 randomized trials, topical Diltiazem was associated with a lower incidence of side effects (relative risk [RR] = 0.48 [0.27–0.86]) and lower incidence of headache (RR = 0.39 [0.24–0.66]) than topical nitroglycerine, with no difference in the healing rate (RR = 1.10 [0.90–1.34]) [53]. In a recent metanalysis of 17 randomized trials [54], topical Diltiazem showed a superior effect compared with nitroglycerin (RR = 1.16 (95% CI = 1.05–1.30); I2 = 18%), with fewer adverse effects (RR = 0.13 (95% CI = 0.04–0.042); I2 = 87%). Similar results were evidenced with the use of topical nifedipine compared with lidocaine (RR = 4.53 (95% CI = 2.99–6.86); I2 = 28%). Regarding recurrence, nifedipine was superior to lidocaine (RR = 0.18 (95% CI = 0.08–0.44); I2 = 31%). Despite the considerable numbers of included trials, the current evidence, due to the studies heterogeneity, supported a grade 1B recommendation.

A single double-blind, randomized, prospective trial on 110 patients with chronic anal fissures compared the safety and efficacy of topical 0.3% nifedipine plus 1.5% lidocaine with topical 1% hydrocortisone plus 1.5% lidocaine. After 6 weeks of treatment, the reported healing rate in the nifedipine group was 95% compared with 16% in the control group, without registering any systemic adverse reaction in patients treated with nifedipine plus lidocaine [55]. Unfortunately, these results have not yet been confirmed by other authors and may have been influenced by the employment of cortisone in the control group. Therefore, according with current evidence, only a weak recommendation may be supported (2B).

Currently, numerous topical ointments with healing effect, including active principles with emollient, moisturizing, antiinflammatory, antibacterial and film-forming properties are commercially available. Overall, considering the scarce relevant literature, no strong recommendation can be made regarding this adjunctive treatment. However, according with the expert panel opinion, the use of these products may be reasonable both in association with topical channel blockers or nitrates for chronic anal fissure associated with anal hypertonia and as exclusive treatment for chronic anal fissures associated with anal hypotonia.

A multicenter randomized controlled trial on 120 patients with chronic anal fissure comparing 53 subjects treated with Propionibacterium extract gel (PeG) (a product with film-forming, anti-inflammatory and healing properties) with 43 subjects treated with glyceryl trinitrate (GTN) ointment, showed no significant difference in the healing rate (53.5% in GTN group vs 56.6% in PeG group: *p* = 0.85) with fewer adverse events in the PeG group. Although these results suggest a potential role of PeG in promote the anal fissure re-epithelialization, the current study has some limitations and its findings should be confirmed by other authors. Consequently, only a weak recommendation may be supported (2B) [56].

### In patients with chronic anal fissure what is the role of botulinum toxin injection?

- *In patients with chronic anal fissure Botulinum toxin injection shows results comparable to topical nitroglycerine as first-line therapy (strong recommendation based on moderate-quality evidence, 1B).*
- *In patients with chronic anal fissure Botulinum toxin injection may be considered as second-line therapy after unsuccessful treatment with topical nitrates (weak recommendation based on low-quality evidence, 2C).*
- *The employment of botulinum toxin injection in patients with chronic anal fissure is limited by the poor diffusion of the procedure and heterogeneity of the adopted injection protocols (experts opinion).*

According with prospective studies and randomized controlled trials, in patients with chronic anal fissure the Botulinum toxin injection has similar results compared to topical nitroglycerine and nifedipine, with an healing rate variable from 43 to 67% [57–60].

This findings were confirmed by a meta-analysis that additionally showed a lower incidence of adverse effects of Botulinum toxin if compared with topical nitrates [61].

Small prospective and retrospective studies suggest that combined use of botulinum toxin and topical nitroglycerine, such as the use of botulinum toxin as second-line therapy after unsuccessful treatment with topical nitroglycerin, may be associated with improvement of healing rate and symptoms relief, providing a chance, in selected cases, to avoiding surgery [62–65].

Unfortunately, both dosing of botulinum toxin and injection technique widely vary among the authors, making the various studies highly heterogeneous in the injections number, injected dose and injected sites [66].

### In patients with chronic anal fissure what is the role of anal dilatation?

- *Uncontrolled anal dilatation is associated with lower healing rate and higher risk of incontinence, if compared with lateral internal sphincterotomy and therefore it can not be recommended (strong recommendation based on moderate-quality evidence, 1B)*
- *Pneumatic balloon dilatation may be offered as treatment option in patients with chronic anal fissure poor responsive to medical therapy and associated with anal hypertonia (weak recommendation based on moderate-quality evidence, 2B)*
- *In the setting of chronic anal fissure associated with anal hypertonia, pneumatic ballon anal dilatation may be preferred to sphincterotomy in multiparous female patients and patients with previous documented sphincter damage or obstetrical injuries (weak recommendation based on moderate-quality evidence, 2B)*

According with a Cochrane review, uncontrolled anal dilatation (digital anal stretch) is at least three times less effective than sphincterotomy and shows a 51% risk of permanent anal incontinence [67].

In order to regulate and standardize the anal dilatation, many techniques have been proposed including a controlled sphincteric dilatation through progression of anal dilators up to a diameter of 48 mm [68], the use of an anal dilator as outpatient treatment [69], the “sphincterolysis” (consisting of rupture of the internal sphincter fibers by firm finger pressure within the anal canal [70]) and the pneumatic balloon dilatation [71–74]. This last procedure includes the insertion in the anal canal of a 40-mm diameter and 60-mm long anal balloon, its rapid insufflation up to a 20 psi pressure (1.4 atm) and the maintaining in situ for five minutes under local anesthesia and mild sedation. A retrospective evaluation of 66 treated patients showed a 94% healing rate with no case of anal incontinence [71]. A prospective evaluation of this technique on 33 patients showed a 94% healing rate with a 6% minor transient fecal incontinence [72]. In a small randomized controlled trial comparing 18 patients treated with pneumatic ballon dilatation and 18 patients treated with local nitroglycerine a significant higher healing rate was found in the dilatation group (94.5% vs. 38.9%) and no one case of postoperative anal incontinence was observed [73]. Subsequently, Renzi et al., in a randomized controlled trial comparing 24 patients undergoing pneumatic balloon anal dilatation with 25 patients undergoing sphincterotomy showed a not significant higher healing rate in the sphincterotomy group (92% vs 83.3%) and a significant lower incontinence rate in the pneumatic ballon dilatation group (0% vs 16%), supporting pneumatic balloon dilatation as a preferable procedure for chronic anal fissure poor responsive to conservative treatment in multiparous female patients, in patients with previous documented sphincter injuries or obstetrical trauma [74]. However, these results arise from analysis of small patients series and have still not been confirmed by a multi-center randomized controlled trial with a large sample size. Therefore, according with these limited available data, only a weak recommendation may be supported (2B).

As regards the use of anal dilation as outpatient treatment, a randomized controlled trial comparing patients with acute anal fissure treated with stool softeners and lidocaine jelly with or without inserting an anal dilator twice daily, showed not significantly differences in the healing rate between the 2 groups, suggesting that the addition of a dilator to the conservative treatment did not decrease the likelihood of surgery [69].

### In patients with chronic anal fissure what is the role of sphincterotomy?

- *Lateral internal sphincterotomy may be offered as a treatment option in patients with chronic anal fissure poor responsive to medical therapy and associated with anal hypertonia (strong recommendation based on high-quality evidence, 1A).*
- *Within this patients group, lateral internal sphincterotomy should be considered as the treatment of choice in the subjects with no clinical complain of fecal incontinence, no previous anorectal operations or trauma, no previous sphincter injuries or obstetrical trauma (strong recommendation based on high-quality evidence, 1A).*
- *Lateral internal sphincterotomy should not be offered to patients with baseline fecal incontinence or with a documented anal sphincter injury or obstetrical trauma (strong recommendation based on high-quality evidence, 1A).*
- *Open and closed techniques of lateral internal sphincterotomy show similar results (strong recommendation based on high-quality evidence, 1A).*

Multiple randomized controlled trials showed that lateral internal sphincterotomy is associated with an higher healing rate if compared with topical nitrates and calcium blockers and one of the reasons may be the poor compliance associated with long-term medical therapy [75–81].

Moreover, other randomized controlled trials confirmed the superiority of lateral internal sphincterotomy compared with botulinum toxin [79–83], manual or pneumatic balloon anal dilatation [74, 84–87] and fissurectomy [88].

Overall, according with these studies, lateral internal sphincterotomy shows an healing rate variable from 88 to 100% with a postoperative fecal incontinence rate ranging from 6 to 30%, based on follow-up intervals up to 6 years. In detail, the post-sphincterotomy anal incontinence mainly occurs as flatus incontinence or soiling, and rarely as major incontinence (for liquid or solid stool). In addition, the postoperative incontinence rate is extremely variable among the authors, as a result of the sphincterotomy extension and the high characteristics variability of patients undergoing surgery. Practically, lateral internal sphincterotomy may be safely offered to the majority of patients with chronic anal fissure poor responsive to medical therapy and associated with anal hypertonia, whereas should not be considered in subjects with baseline fecal incontinence, patients who have undergone previous anorectal surgery and with documented anal sphincter injuries or obstetrical trauma [89–91].

In the light of a Cochrane analysis of 5 studies including 336 patients, there is no statistical difference with regard to fissure healing (OR 1.00, 95% CI, 0.40–2.48) and incontinence to flatus (OR 0.87; 95% CI, 0.41–1.83) between open and closed techniques of lateral internal sphincterotomy [67], even if, in a randomized trial, the open technique was associated with significantly higher postoperative pain scores and higher delayed healing rate if compared to closed one [92].

According to a retrospective comparative study, the excision of hypertrophied anal papilla and fibrous anal polyp after sphincterotomy has been associated with lower pain and irritation during defecation (*P* = 0.0011), lower pricking or foreign body sensation in the anus (*P* = 0.0006) and lower pruritus or wetness around the anal verge (*P* = 0.0008) [93]. However these results have still not been confirmed by a randomized controlled trial.

### What is the adequate extension of sphincterotomy?

- *A safe lateral internal sphincterotomy should be confined below the level of the dentate line (strong recommendation based on moderate-quality evidence, 1B)*
- *Lateral internal sphincterotomy tailored to the length of the fissure is equally effective and safer than conventional sphincterotomy extended to the dentate line (strong recommendation based high-quality evidence, 1A)*
- *In female patients the ideal extension of the internal sphincter division should be between 5 and 9 mm of the muscle, without ever exceeding 10 mm (strong recommendation based on low-quality evidence, 1C)*

The internal sphincterotomy was first described in the early 1800s and performed at the level of posterior commissure, in the fissure bed, and subsequently proposed in 1930 by Gabriel, in association with fissure excision [94]. This technique, although associated with an high healing rate, was frequently followed by the “keyhole” deformity resulting in fecal soiling in up to a third of patients [95]. For these reasons the posterior sphincterotomy has gradually fallen disused and, now, it is rarely adopted in most specialized centers.

Lateral sphincterotomy was first proposed in 1951 by Eisenhammer who, in his initial description, recommended four-fifths to total division of the lateral internal sphincter [96]. However, this technique was quickly abandoned, as proving to be a cause of fecal incontinence. Eisenhammer revised the technique in 1959, stating that a lesser division to the dentate line was safer and adequate in many cases, giving rise to the “dogma of dentate line” [97]. Afterwards, in 1969, Notaras proposed the technique of closed subcutaneous lateral sphincterotomy extended “just above the dentate line” [98].

The lateral sphincterotomy extended to the level of dentate line, although safer than the posterior and total sphincter division, was however associated with a not negligible prevalence of postoperative continence disorders, especially in female patients, probably due to their particular anal canal conformation. In this regard, basing on a prospective endosonographic evaluation of 15 females undergoing sphincterotomy, Sultan et al. showed that in most females, due to their shorter anal canal, the division of the internal anal sphincter up to dentate line was more extensive than intended and that this internal sphincterotomy may compromise sphincter function and precipitate anal incontinence, particularly in the presence of other sphincter defects [99]. Similarly, in another endosonographic and manometric study comparing 13 patients with anal incontinence after lateral internal sphincterotomy with 13 continent controls who underwent the same operation, fecal incontinence was directly related to the length of the sphincterotomy [100].

Basing on these considerations and findings, since the 1990s, more conservative sphincterotomies have been proposed.

Pernikoff et al., in a large series of sphincteromies performed “distal to dentate line”, reported a 98% success rate with 8% fecal incontinence rate over the long term [101].

Littlejohn et al. proposed a “tailored” sphincterotomy, defined as sphincterotomy limited in extent to the apex of the fissure, achieving, in a large retrospective series of 287 patients during a 30-year period, a high success rate (99%) with only 1.4% rate of flatus incontinence, and no patients experiencing incontinence to stool [102]. These findings were substantially confirmed by two randomized controlled trials comparing conventional with tailored sphincterotomy [103, 104].

Garcea et al. described in 65 patients a conservative lateral sphincterotomy extended for no more than 5 mm of the muscle, reporting, at a mean 6.9 weeks follow-up, a 90% success rate with a flatus incontinence rate of 3.3% and liquid incontinence rate of 1.7% [105].

A prospective evaluation with three-dimensional endoanal ultrasound and Wexner incontinence score of 31 female patients undergone internal sphincterotomy, showed that the percentage of patients with a continence score of 0 was significantly higher in patients in whom sphincter division was less than 25% in comparison with patients with a division of 25% or more. These results suggested that in female patients the safe extent of division should be less than 25% of the total sphincter length, corresponding, in the study population, to less than 1 cm of the muscle [106].

According with these results, a prospective study evaluating 32 female patients with perfect anal continence undergoing a “minimal” lateral internal sphincterotomy extended for about 20% of total sphincter length (corresponding to an extension of sphincter division between 5 and 8 mm), showed, at 12 months follow-up, a 100% success rate with no case of worsening incontinence [107].

In conclusion, the available literature seems to support with high-quality evidence the tailored sphincterotomy extended to the apex of the fissure instead of the conventional sphincterotomy in the surgical treatment of chronic anal fissure. Moreover, according with prospective studies with small sample size, in female patients the ideal sphincterotomy should be between 5 and 9 mm, without ever exceeding 10 mm.

### In patients with chronic anal fissure what is the role of fissurotomy and fissurectomy?

- *According with the scarse available literature, no recommendation can be made regarding the employment of fissurotomy in the treatment fo chronic anal fissure.*
- *Fissurectomy is inferior to lateral internal sphincteromy in the treatment of chronic anal fissure associated with internal anal sphincter hypertonicity (strong recommendation based on high-quality evidence, 1A)*
- *Fissurectomy may be considered in patients with chronic anal fissure associated with abscess or fistula and normotonic internal anal sphincter (weak recommendation based on low-quality evidence, 2C)*

Fissurotomy consists in the deroofing of the subcutaneous tract extending caudally to the anal fissure, eventually associated with the excision of the residual sentinel tag. It represents an old procedure, resumed by Pelta et al. which described it in a series of 109 patients undergoing surgery for chronic anal fissure [108]. In this study the authors, using a narrow-gauge, hooked probe, reported a constant, midline subcutaneous tract extending from the caudal aspect of the fissure. Therefore, laying open this tract, without the need of a contextual sphincterotomy, the authors obtained, at 12 months median follow-up, a very high success rate (98.2%) with no change in continence in all the patients. These intra-operative findings are discordant with those of other studies that showed an associated sinus or fistula in a limited percentage of patients with chronic anal fissure [19–21]. Moreover, in this series, the fissurotomy was performed employing an anorectal surgical speculum, raising the question of whether the speculum anal dilatation could have affected the reported healing rate [109]. Anyway, the impressive favorable results obtained with fissurotomy in the study of Pelta et al. have still not been confirmed by other authors. Therefore, according with the scarse available literature, no recommendation can be made regarding the employment of fissurotomy in the treatment fo chronic anal fissure.

Fissurectomy includes excision of the fibrotic edge of the fissure, curettage of its base, and excision of the sentinel pile and ⁄ or anal papilla if present. Differently from other techniques, fissurectomy allows to obtain an histological examination of the fissure. The resulting defect may be left open and healing by secondary intention or surfaced by an anoplasty, advancing a circumcised area of perianal skin proximally into the anal canal (cutaneous advancement flap).

Concerning the isolated fissurectomy, although some retrospective and prospective observational studies reported an high healing rate (> 90%) with minimal influence on anal continence [110–113], two randomized controlled trials showed the superiority of lateral internal sphincterotomy over fissurectomy in terms of patients postoperative satisfaction and continence [88, 114]. Similarly, a Cochrane Collaboration meta-analysis found lateral internal sphincterotomy to be associated with higher healing rate and similar risk of postoperative anal incontinence, if compared with fissurectomy [67].

The combination of fissurectomy with “chemical sphincterotomy” has been associated with high healing rate (between 80 to 100%) and a decreased risk of incontinence [115, 116]. However these results should be interpreted with caution due to the low level of evidence of these studies. Notewhorthy, the combination of fissurectomy with an internal sphincterotomy in the bed of the fissure, although may be useful in case of associated local sepsis, is complicated in a third of patients by keyhole deformity of the anal canal with fecal soiling [94, 95].

Substantially, according with literature, lateral internal sphincteromy should be preferred to fissurectomy as surgical option for patients with chronic anal fissure associated with internal anal sphincter hypertonia. Anyway, fissurectomy, achieving the replacing of poor quality tissues that poorly heal with a clean wound that may quickly heal, may be considered in patients with chronic anal fissure associated with local sepsis and normotonic internal anal sphincter.

The employment of fissurectomy alone for chronic anal fissure even in patients with anal hypertonia is based on the theory that anal fissure chronicity only represents the clinical expression of an underlying sepsis and that the internal anal sphincter spasm, when present, is only an epiphenomenon of the infection [20, 108]. However, the above-mentioned available literature seems not support this etiopathogenetic hypothesis.

### In patients with chronic anal fissure what is the role of anocutaneous flap?

- *In light of the low postoperative risk of incontinence, anocutaneous flap may be considered as an alternative surgical option in patients with chronic anal fissure and high risk of incontinence after sphincterotomy (low anal resting pressure, previous anal surgery or trauma, previous documented sphincter injuries or obstetrical trauma) (weak recommendation based on moderate-quality evidence, 2B).*
- *The addition of anocutaneous flap to sphincterotomy or botulinum toxin injection may decrease postoperative pain, improve healing rate and reduce postoperative incontinence rate (weak recommendation based on low-quality evidence, 2C).*

The anocutaneous flap coverage (with dermal V-Y or house flap) of the defect resulting from fissurectomy has been associated with high fissure healing rates (81%–100%) and low rates of minor incontinence (0%–6%) [117–120].

In a prospective randomized study comparing flaps (*n* = 50) with sphincterotomy (*n* = 50) the fecal incontinence rate was significantly higher in the sphincterotomy group (2.5% vs 17%: *p* = 0.01) [121].

According to a systematic review and metanalysis, anal advancement flap was associated with a significantly lower rate of anal incontinence compared to lateral internal sphincterotomy (OR = 0.06, 95% CI = 0.01 to 0.36, *p* = 0.002). However, there were no statistically significant differences in unhealed fissure (OR = 2.21, 95% CI = 0.25 to 19.33, *p* = 0.47) or wound complication rates (OR = 1.41, 95% CI = 0.50 to 4.99 *p* = 0.51) between the two procedures [122].

Three observational studies showed that combination of botulinum toxin injection and flap was associated with 86.7% to 100% healing rate and a with negligible postoperative incontinence rate [120, 123, 124].

A randomized controlled trial comparing 50 patients undergoing lateral internal sphincterotomy (group 1), with 50 patients undergoing isolated V-Y advancement flap (group 2) and 50 patients undergoing lateral internal sphincterotomy combined with V-Y advancement flap (group 3) showed, at 1 year follow-up, a significantly higher success rate in the third group if compared with the other 2 groups (94% vs 84% in group 1 and 48% in group 2: *p* = 0.001) associated with a significantly lower incontinence rate in patients undergoing combined sphincterotomy and flap if compared with those undergoing sphincterotomy alone (2% vs 14%: *p* = 0.03) [125]. These results suggest that addition of the flap to sphincterotomy may improve healing rate and possibly reduce fecal incontinence rate.

The current literature lacks of prospective-randomized studies comparing isolated fissurectomy with combined fissurectomy and flap in treatment of chronic anal fissure. Generally, in clinical practice, isolated fissurectomy is preferred in case of infected anal fissures whereas the addition of flap is considered in the case of non-infected fissures. In a series of 257 patients with chronic anal fissure treated with combined fissurectomy and anoplasty in 83% of cases and with isolated fissurectomy in 17%, anoplasty did not impact any result [113]. Additionally, a recent retrospective study including 226 patients with non infected posterior anal fissures and comparing 182 isolated fissurotomies with 44 combined fissurectomies and advancement flap anoplasty, showed no difference in time to relief of pain, time to disappearance of bleeding and time to healing between the two groups [126], therefore questioning the effectiveness of an associated flap even in non-infected fissures. However, these data should be confirmed by other authors and larger prospective-randomized studies are needed to address this question [127–130].

### In patients with chronic anal fissure, what is the role of tibial nerve stimulation?

- *Percutaneous tibial nerve stimulation may be considered as a potential altenative treatment for chronic anal fissure (weak recommendation based on low-quality evidence, 2C).*
- *Percutaneous tibial nerve stimulation may be considered as a potential therapeutic option for chronic anal fissure resistant to other conservative measures in patients unfit for surgery or who refuse surgical treatment (experts opinion)*

Recently, neuromodulation has been proposed as alternative sphincter-saving procedure for treatment of chronic anal fissure [131–133]. Although the exact mechanim of action has not yet been fully clarified, the activation of the sacral neural pathways can lead to increased anal perfusion, activation of epithelial cells and keratinocytes, migration of fibroblasts and macrophages and deposition of collagen, resulting in improved and faster mucosal healing [134].

In order to avoid the need for surgical implantation of sacral neuromodulation unit, percutaneous stimulation of the posterior tibial nerve (PTNS) was proposed [135–139].

Few small-sample observational studies and 2 randomized controlled trials with some limitations showed promising results of PTNS in treatment of chronic anal fissure, with low mordibity rate [135–139].

A systematic review of 5 studies including 102 patients, estimated a pooled recurrence rate of 19% (16/84) with considerably reduced post-interventional pain scores, a 2-month healing rate of 72% (18/25) and 73.6% of patients symptom-free at 6 months [140]. However, the evaluated studies showed considerable limitations related to the small sample size, short-term follow-up, study design and heterogeneity in the neuromodulation technique and setting.

Interestingly, a randomized controlled trial comparing 1 year anal fissure recurrence between lateral sphincterotomy and percutaneous posterior tibial nerve stimulation showed the clear superiority of surgery, with recurrence rates of 2.7% and 40.7% in sphincterotomy and neuromodulation group, respectively [136].

In view of all this, the Expert Panel opted for considering percutaneous tibial nerve stimulation as a potential therapeutic option for chronic anal fissure resistant to other conservative measures and in patients unfit for surgery or who refuse surgical treatment.

## Diagnostic and therapeutic algorithm for typical anal fissure

**Fig. 1:**
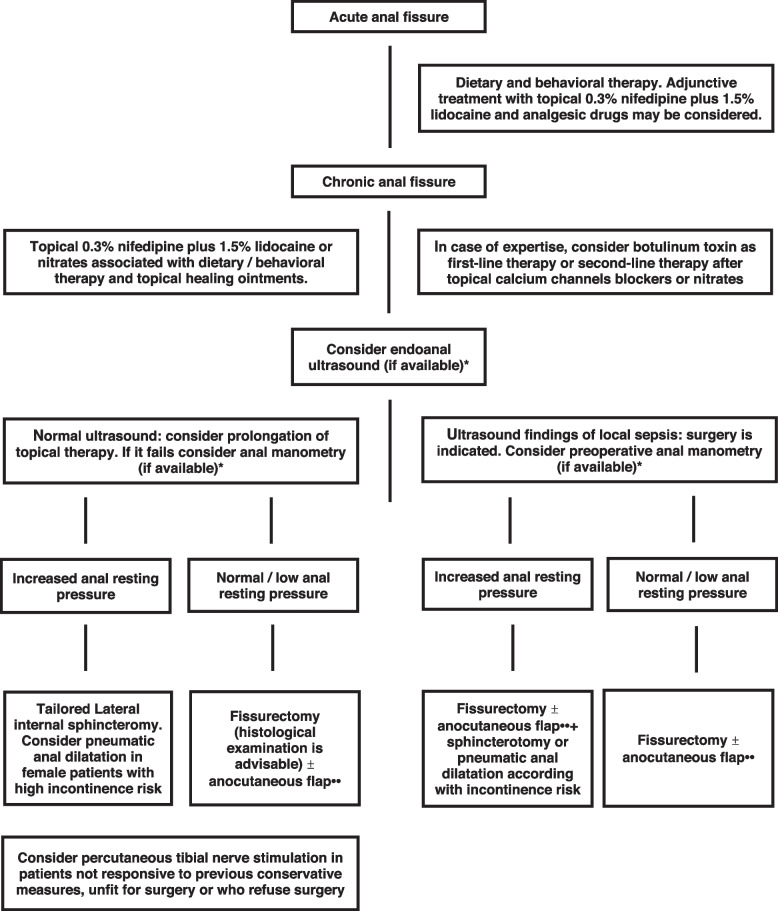
Diagnostic and therapeutic algorithm for typical anal fissure. *According with the low grade of evidence supporting the preoperative morphological and functional investigations, the choice to perform both endoanal ultrasound and anal manometry should be considered optional in the clinical practice. Therefore, the evaluation of the anal tone may be carried out by digital examination and the detection of an associated local sepsis may be performed during surgery. **The choice to perform an anocutaneous flap may be based on surgeon preference and on intraoperative findings. Particularly, the addition of a flap should be carefully considered in presence of gross local infection. In these cases an isolated fissurectomy should be reasonably preferred

From previous statements a diagnostic and therapeutic algorithm may arise (Fig. 1), including the possibility to performe the morphological (endoanal ultrasound) and functional (anal manometry) investigations discussed in the guidelines. Naturally, considering the low grade of evidence supporting the employment of these investigations, the current algorithm should be considered optional and the choice of the more appropriate diagnostic-therapeutic planning should be left to single physician, according to the specific clinical case and the available resources.

In patients with acute anal fissure the conservative treatment with dietary and behavioral norms is indicated. The adjunctive treatment with common analgesic drugs is reasonable whereas, the employment of topical 0.3% nifedipine plus 1.5% lidocaine may be considered in the subjects with poor adherence to hygienic-dietary medical prescriptions.

In the chronic phase of disease, the conservative treatment with topical 0.3% nifedipine plus 1.5% lidocaine or nitrates may represent the first-line therapy, in association with the common dietary-behavioral norms and ointments with film-forming, anti-inflammatory and healing properties. As alternative, the botulinum toxin injection may be performed in experienced centers.

In case of first-line treatment failure (after at least 3 weeks therapy), endoanal ultrasound may be considered, if available.

If endoanal ultrasound shows findings of local sepsis, surgical treatment is recommended and preoperative manometry may be considered. In case of normal or low anal resting pressure, fissurectomy, eventually combined with anocutaneous flap, may be indicated whereas, in case of high anal resting pressure, fissurectomy and anocutaneous flap may be combined with sphincterotomy or pneumatic anal dilatation, according with the individual patient incontinence risk.

In presence of normal ultrasound findings, a prolongation of topical medical therapy may be considered, modulating the treatment by addition or replacement of myorelaxant and healing active principles. If this further trial fails, surgery is indicated and preoperative manometry may be considered. In case of high anal resting pressure, tailored lateral internal sphincterotomy or pneumatic anal dilatation may be offered, according with the patients risk of incontinence. In case of normal or low resting pressure, a fissure excision (eventually combined with anocutaneous flap) with histological examination is advisable. In all the other cases, the fissure histological examination may be considered optional. Finally, in patients with not infected chronic anal fissure resistant to other conservative measures, unfit for surgery or who refuse surgical treatment, percutaneous tibial nerve stimulation may be considered as a potential treatment option.

## Data Availability

All data generated or analysed during this study are included in this published article.

## Abbreviations

SIUCP: Società Italiana Unitaria di Colonproctologia
PICO: Patients, Intervention, Comparison, Outcome
GRADE: Grading of Recommendations, Assessment, Development, and Evaluations
GTN: Glyceryl Trinitrate
PeG: Propionibacterium extract gel
PTNS: Percutaneous tibial nerve stimulation
